# Clinicopathological features of idiopathic membranous nephropathy combined with IgA nephropathy: a retrospective analysis of 9 cases

**DOI:** 10.1186/s13000-016-0538-7

**Published:** 2016-09-13

**Authors:** Ruimin Hu, Guolan Xing, Huijuan Wu, Zhigang Zhang

**Affiliations:** 1 Department of Pathology, School of Basic Medical Sciences, Fudan University, 138, Yixueyuan Road, Xuhui District, Shanghai, 200032 People’s Republic of China; 2 Department of Nephrology, The First Affiliated Hospital of Zhengzhou University, 1, Jianshe Road East, Erqi District, Zhengzhou, Henan 450052 People’s Republic of China

**Keywords:** Idiopathic membranous nephropathy, IgA nephropathy, PLA2R

## Abstract

**Background:**

The concomitant presence of idiopathic membranous nephropathy and IgA nephropathy is rare. Here, we report 9 cases of phospholipase-A2-receptor (PLA2R) positive idiopathic membranous nephritis combined with IgA nephropathy, while reviewing publications regarding the pathological characteristics of this glomerolonephritis complication.

**Case presentation:**

Nine cases of renal biopsy tissues were retrospectively reviewed, including the clinicopathological features, the results of the immunofluorescence assays, and the electron microscopic examination. The patients mainly presented proteinuria and microscopic hematuria, and the serum anti-PLA2R was detected as positive in all of the patients. Histologically, a wide thickening of the glomerular basement membrane was observed in each of the 9 cases. Additionally, there existed mild hyperplasia in the mesangial cell and the matrix of the mesangial area. Immunofluorescence assays showed prominent glomerular granular staining on the glomerular capillary loops for IgG (++/+++), IgG4 (++/++++), and PLA2R (+/++). In addition, moderate IgA positive stains were focally or sparsely limited to the mesangial areas. Electron microscopy revealed subepithelial and mesangial electron-dense deposits.

**Conclusions:**

The results from the case analyses indicated that idiopathic membranous nephropathy combined with IgA nephropathy possess the clinicopathological features found in both components. It is suggested that serum anti-PLA2R and tissue PLA2R are important biomarkers that can assist in the diagnosis of idiopathic membranous nephropathy associated with IgA nephropathy.

## Background

Membranous nephropathy (MN) and IgA nephropathy (IgAN) are two distinct glomerular diseases. The most common cause of nephrotic syndrome in adults can be attributed to MN, in which approximately 70 % of this disease can be categorized as idiopathic MN (also referred to as primary MN). In Chinese as well as other Asian populations, IgAN has become a major pathological type of glomerular disease. Furthermore, it was estimated that IgAN accounts for 45.2 to 58.2 % of primary glomerular diseases in China [1–3]. Recently, an increasing number of reports have emerged in regards to IgAN combined with other renal diseases [1, 4]. However, studies involving the concomitant presence of idiopathic MN and IgAN are rare. The few reports available from studies conducted around the world rely heavily on immunofluorescence and electron microscopic examination to identify patients with combined idiopathic MN and IgAN. These data showed subepithelial and mesangial electron-dense deposition (EDD) in which the immunofluorescence analysis conducted on the patient samples was positive for both IgG and IgA [5–10]. However, it may bring some confusion in diagnosing these complications by immunofluorescence and electron microscopic examination, because some secondary MN also showed mesangial EDD in additional to subepithelial deposition, while the IgAN sometime have minimal subepithelial EDD with mesangial massive deposition.

Recently, serum anti-phospholipase-A2-receptor (PLA2R) antibodies were found to be the main serum auto-antibodies presented in idiopathic MN patients. Furthermore, previous reports have demonstrated that M-type PLA2R is a specific marker for idiopathic MN [11, 12]. It has been shown that PLA2R is hardly detected in secondary MN and other glomerular diseases [13, 14]. Therefore, the test of anti-PLA2R as a sensitive biomarker may provide a novel diagnostic tool for idiopathic MN, and for the differentiation of secondary MN [11, 15, 16]. This biomarker also provides a more definitive evidence for detecting idiopathic MN combined with IgAN in patients. In this article, we report 9 cases of PLA2R-positive idiopathic MN combined with IgAN, in addition to a review that discusses and compares our findings with previous publications.

## Materials and methods

### Patients

Between January 2013 and December 2015, 9 cases of patients with MN combined with IgAN were selected from the nephrosis laboratory of the Department of Pathology, School of Basic Medical Sciences, Fudan University, Shanghai, China and The First Affiliated Hospital of Zhengzhou University, Zhengzhou, China.

### Data collection

These cases are comprised of patients whose renal biopsies showed glomerular predominant IgG and PLA2R deposits with more or less mesangial IgA deposition, excluding the cases of differentiating renal diseases such as lupus nephritis, HBV associated nephritis and antineutrophil cytoplasmic autoantibody (ANCA)-associated crescentic glomerular nephritis (GN) with mesangial IgA deposits. The medical records of the patients were reviewed for age, gender, type and degree of hematuria, urinary protein excretion (g/24 h), and serum creatinine at the time of biopsy.

### Biopsy evaluation

Histological slides from each biopsy were stained with hematoxylin-eosin, periodic acid-Schiff, silver methenamine, and masson’s trichrome. In addition, immunofluorescent staining of immunoglobulins, complement, and PLA2R were conducted. These stained samples as well as all of the transmission electron micrographs taken at the time of initial biopsy evaluation were reviewed by two renal pathologists (Dr. Wu and Dr. Zhang).

## Case presentation

The clinical and serologic features of the 9 patients, 4 females and 5males, were summarized in Table 1. The mean age was 38.3 ± 7.4 years (range 25–48 years). The patients had no history of renal disease and bacterial infection prior to the occurrence of renal complications. The patients had nephrotic syndrome and the mean proteinuria level was 4.82 ± 3.33 g/24 h (range 1.79–11.76 g/24 h). 7 of the 9 patients had microscopic hematuria, whereas all patients had no gross hematuria. The mean serum creatinine at the time of biopsy was 68.67 ± 17.55 μmol/L (range 49–93 μmol/L). All patients had increased serum PLA2R antibody prominently with a mean level of 57.39 ± 47.89 RU/mL (range 24.6–177.1RU/mL), while the serum IgA and complement C3 were closely distributed within the normal range in these 9 patients. 8 of the 9 cases had no hypertension history, and only case 2 had hypertension for more than 10 years (92–100/136–145 mmHg controlled by extended release nifedipine tablets). The ANA,ANCA and HBV markers detected at the time of biopsy were negative for each of the 9 cases.

**Table 1:** Summary of the clinical and serological features of renal biopsies from 9 patients

	Ca.1	Ca.2	Ca.3	Ca.4	Ca.5	Ca.6	Ca.7	Ca.8	Ca.9
Age	41	42	28	42	36	25	41	48	42
Sex	F	F	M	M	M	M	F	F	M
Prodromal infection history	Non	Non	Non	Non	Non	Non	Non	Non	Non
Family history of kidney diseases	Non	Non	Non	Non	Non	Non	Non	Non	Non
Gross hematuria	Non	Non	Non	Non	Non	Non	Non	Non	Non
Hypertension	Non	>10ys	Non	Non	Non	Non	Non	Non	Non
Microscopic hematuria(/μL)	37	8.58	29	27	15.18	45	0	40	0
Proteinuria	+++	++	+++	+++	++++	+++	++	+++	+++
24TP(g/24 h)	2.97	1.79	2.28	7.61	11.76	2.59	2.4	6.42	5.52
Serum creatinine(μmol/L)	49	68	90	64	93	66	52	49	87
Serum ALB(g/L)	25.8	32.8	34.6	21.2	20.6	44.9	36.1	23.5	27.4
Serum IgA(g/L)	1.12	3.32	1.36	2.58	1.84	3.59	1.11	4.8	2.44
Serum C3(g/L)	0.87	1.45	0.93	1.34	1.05	1.08	0.95	1.32	1.01
Serum anti-PLA2R(RU/mL)	69.1	34.6	31.5	53.4	177.1	27	24.6	32.9	66.3
Serum autoantibodies	-	-	-	-	-	-	-	-	-
Serum HBV markers	-	-	-	-	-	-	-	-	-
ANCA	-	-	-	-	-	-	-	-	-

Note: *Ca.* case, *F* female, *M* male

Table 2 lists the summary of the histopathological features of the renal biopsies conducted within each of the 9 cases. The biopsies showed a predominantly diffuse thickening of the basement membrane of glomerular capillary walls. Mildly mesangial hyperplasia was observed within 4–5 mesangial cells as well as a small portion of the matrix in all 9 cases. Moreover, focal glomerular tuft necrosis or crescents were absent, while mild chronic interstitial inflammation was present in the patient samples.

**Table 2:** Summary of the histopathological features of renal biopsies of the 9 patients

	Ca.1	Ca.2	Ca.3	Ca.4	Ca.5	Ca.6	Ca.7	Ca.8	Ca.9
LM									
%globally sclerotic glomeruli	0/30	1/23	0/18	0/33	0/24	1/18	1/38	0/20	1/31
%segmentally sclerotic glomeruli	0/30	0/23	0/18	0/33	0/24	0/18	1/38	0/20	1/31
Mesangial cell proliferation	+	+	+	+	+	+	+	-	+
Mesangial matrix hyperplasia	+	+	+	+	+	+	+	+	+
Thickening of the capillary walls	++	+	++	++	++	++	++	++	++
Interstitial inflammatory	+	+	+	+	±	-	+	-	-
Focal tubular atrophy	+	+	+	+	-	-	-	-	+
IF									
IgG	+++	++	+++	+++	+++	++	++	++	+++
IgA	++(f)	+	++	+(f)	++	++	++	++	++
IgG1	+/++	±	++	-	++	+	-	+	-
IgG2	±	+	-	-	±	-	±	±	+
IgG3	±	+/++	-	+	+	-	±	±	-
IgG4	++	++	++++	++	+++	++	++	++	++
PLA2R	+	+	+	++	+/++	+	+	+	+
C3	+/++	+	++	+/++	+++	+	+/++	+	++
EM									
Subepithelium EDD	++	++	++	++	++	++	+++	++	++
Mesangial area EDD	++	+	+	+	+	+	++	+	+
Extensive processes effacement	++	++	++	++	+/++	+	++	++	++
Mesangial matrix hyperplasia	+	-	+	+	-	-	+	-	+

Note: *Ca.* case, *f* focal

**Fig. 1:**
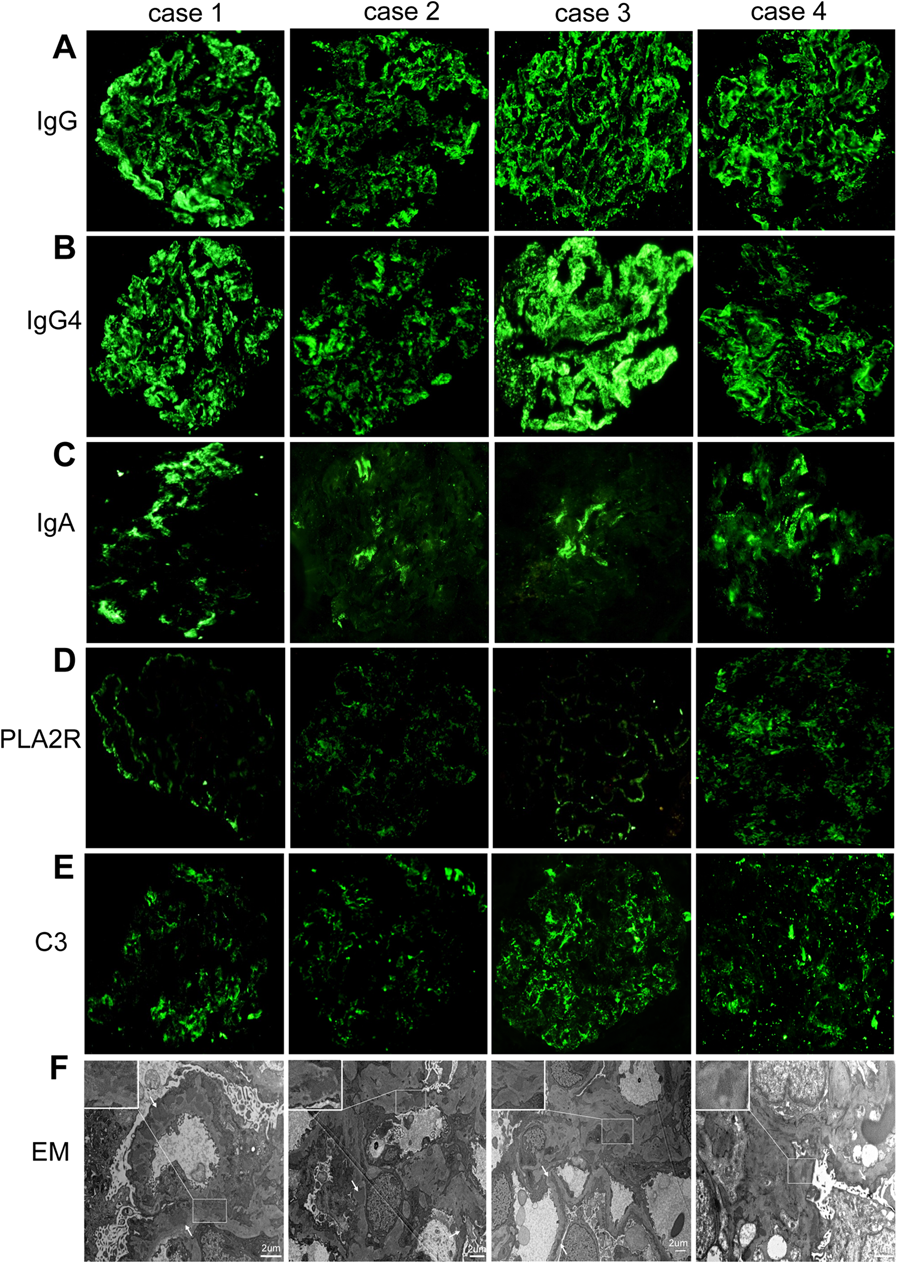
Cases 1–4 immunofluorescence for immunoglobulins and EDD detected by electron microscopy. Immunofluorescence in cases 1–4 for **a** IgG, **b** IgG4, **c** IgA, **d** PLA2R and **e** C3. **f** The EDD detected by electron microscopy in case 1–4. White arrows, subepithelial deposits; Inset: boxed mesangial deposits

In the immunofluorescence staining, each of the cases showed prominent glomerular granular staining on glomerular capillary loops for IgG (++/+++), IgG4 (++/++++), and PLA2R (+/++); C3 (+/+++) also displayed granular staining on glomerular capillary loops. IgA (+/++) positive staining were focally or sparsely limited to the mesangial areas (Fig. 1a–e).

For the electron microscopy, granular EDD were present in the subepithelium in 9 patients, in addition to irregular thickening of the glomerular basement membrane and segmental spikes. The extensive fusion of epithelial foot processes was also observed. Mild, focal proliferation of mesangial cells and the matrix were noted (Fig. 1f).

## Discussion and conclusion

We examined the clinical and morphological features of samples collected from each of the 9 cases of idiopathic MN combined with IgAN. This complication of GN was defined morphologically, with emphasis on the presence of the PLA2R antibody in serum and PLA2R expression in glomeruli, rather than on the basis of the immunofluorescence assay and transmission electron microscopy.

MN and IgAN are both glomerular diseases mediated by immune complex deposition. The depositing site of the immune complex is different in these two forms of GN due to the diverse mechanisms associated with immune complex formation. These mechanisms are influenced by many factors such as the relative molecular weight and the volume and charge of the immune complex, which may be the cause of the different clinical manifestations and pathological morphology of the two renal diseases. It is found that idiopathic MN with IgAN complications combine the clinical and pathological manifestations of these two types of glomerular diseases. Previous reports regarding patients presenting idiopathic MN with IgAN show that the average age of the patients was 40 years old. Furthermore, the patients in those studies had proteinuria, of which 50 % exhibited heavy proteinuria with nephrotic syndrome. 90 % of the patients examined in these reports presented microscopic hematuria, and 30 % of these patients presented hypertension [6–10]. In this group, the clinical and pathological features of the 9 cases examined were similar to that found in previous reports. These findings are consistent with the clinical characteristics of MN and IgAN. However, the differences noted in the previous reports and the current study are that the age of renal disease onset in the 9 cases is younger than that in the simple idiopathic MN, and that the deposition of IgA EDD was lesser compared to IgG EDD in patients of MN combined with IgAN.

In the present study, the immunofluorescence staining showed granular positive IgG along the capillary loop, while IgA was shown to be irregularly positive in the mesangial area. Meanwhile, the EDD of the subepithelial and mesangial area were found using electron microscopy. The mixed immunoglobulins deposition phenomenon is common in many secondary glomerulonephritis, which may cause confusion for diagnosis and differentiation. Wang et al. reported that use of the colloidal gold-immune electron microscopy technique displayed an IgG positive subepithelial deposition, while the anti-IgA was positive in the mesangial deposition, confirming the different location of IgG and IgA in the complication [10].

Idiopathic MN is recognized as an autoimmune disease [17]. Recently, progress has been made in understanding the pathogens of idiopathic MN with the finding of M-type PLA2R as the target antigen of podocytes in patients with idiopathic MN [11, 12]. It has been revealed that a majority of patients with idiopathic MN were found to possess anti-PLA2R autoantibodies in circulation, tissue PLA2R expression in their biopsy kidney tissue, and that the level of autoantibody correlates with the level of proteinuria [11, 18]. Moreover, Beck et al. along with other investigators showed that serum anti-PLA2R or tissue PLA2R is high detect ability in idiopathic MN, but both are rarely detected in secondary MN [15]. Hoxha et al. found that a high ratio of PLA2R positive in the stained glomeruli of patients with idiopathic MN tightly correlates with the presence of serum PLA2R autoantibodies, while this correlation is lower in secondary MN with respect to PLA2R staining [16]. This method of detection may help discriminate between idiopathic MN from secondary MN. In the 9 cases presented herein, we observed increased serum anti-PLA2R, and glomerular PLA2R positive immunofluorescence staining, associated with IgG subepithelial deposits,and immunofluorescence positive of mesangial IgA and mesangial EDD deposition. Therefore, these patients were confirmed as idiopathic MN combined with IgAN. It is suggested that PLA2R determination is an important assistant biomarker for the diagnosis of idiopathic MN associated with IgAN.

Of note, the 9 cases of idiopathic MN combined with IgAN presented herein had predominant IgG4 and PLA2R co-localized within the subepithelial immune deposits. IgG subtype analysis has been suggested that IgG4-predominant staining is mainly associated with idiopathic MN, whereas IgG1, IgG2, and IgG3 predominate in the deposits of secondary MN [11, 19–23]. Preliminary studies suggest that there may be a direct interaction of anti-PLA2R-IgG4 with the PLA2R molecule, and this interaction activates complement by binding the Fc portion of IgG4 [24]. Furthermore, anti-PLA2R-IgG4 has been described as a predominant circulating IgG subclass in idiopathic MN. The presence of these combined molecules seem to be specific (89 %) to idiopathic MN [13, 25, 26] and can be utilized as supportive data to exclude the presence of secondary MN [13]. Herein, the analysis of the presented cases reveal the significant implications for the diagnosis of idiopathic MN complicated with other renal diseases.

## Abbreviations

ANCA: Anti-neutrophil cytoplasmic autoantibody
EDD: Electron-dense deposition
GN: Glomerular nephritis
IgAN: IgA nephropathy
MN: Membranous nephropathy.
